# Physical activity types and risk of dementia in community-dwelling older people: the Three-City cohort

**DOI:** 10.1186/s12877-020-01538-3

**Published:** 2020-04-10

**Authors:** Caroline Dupré, Bienvenu Bongue, Catherine Helmer, Jean François Dartigues, David Hupin, Frédéric Roche, Claudine Berr, Isabelle Carrière

**Affiliations:** 1 CETAF, Saint-Etienne, France; 2 Laboratoire SNA-EPIS, University Jean Monnet, Saint-Etienne, France; 3 Univ. Bordeaux, Inserm, Bordeaux Population Health Research Center, UMR 1219, CIC 1401-EC, F-33000 Bordeaux, France; 4 Univ. Montpellier, Inserm U1061, Neuropsychiatry: epidemiological and clinical research, PSNREC, 39 avenue Charles Flahault, BP 34493, 34093 Montpellier cedex 05, France

**Keywords:** Dementia, Physical activity, Cohort, Dose-response effect

## Abstract

**Background:**

Physical activity may decrease the risk of dementia; however, previous cohort studies seldom investigated the different types of physical activity and household activities. Our objective was to analyze the links between two physical activity types and dementia in older people.

**Methods:**

The study used data from the prospective observational Three-city cohort and included 1550 community-dwelling individuals aged 72 to 87 without dementia at baseline. Physical activity was assessed with the Voorrips questionnaire. Two sub-scores were calculated to assess household/transportation activities and leisure/sport activities. Restricted cubic spline and proportional hazard Cox models were used to estimate the non-linear exposure-response curve for the dementia risk and the appropriate activity level thresholds. Models were adjusted for possible confounders, including socio-demographic variables, comorbidities, depressive symptoms and APOE genotype.

**Results:**

The median age was 80 years, and 63.6% of participants were women. After a median follow-up of 4.6 years, dementia was diagnosed in 117 participants (7.6%). An inverse J-shaped association was found between household/transportation physical activity sub-score and dementia risk, which means that the risk is lowest for the moderately high values and then re-increases slightly for the highest values. The results remained significant when this sub-score was categorized in three classes (low, moderate, and high), with hazard ratios (95% confidence interval) of 0.55 (0.35–0.87) and 0.62 (0.38–1.01) for moderate and high activity levels, respectively. No significant effect was found for leisure/sport activities.

**Conclusions:**

The 5-year risk of dementia was significantly and negatively associated with the household/transportation activity level, but not with the leisure and sport activity sub-score. This highlights the importance of considering all physical activity types in 72 years or older people.

## Background

Dementia affects 47 million people worldwide, and about 5% of the elderly population. According to recent projections, the number of patients with dementia will reach 132 million in 2050, and globally nearly 9.9 million people develop dementia each year [1]. In addition to age, the presence of the e4 allele of the apolipoprotein E (*APOE*) gene increases the risk of dementia, and may strengthen the effect of other risk factors. Modifiable risk factors include education level, obesity, hypertension, diabetes, diet, and physical activity (PA) [2].

Several meta-analyses and systematic reviews have investigated PA association with late-life cognitive disorders [3] and dementia [4–6]. Most, but not all observational studies found that PA was associated with a decreased risk of dementia [7–9]. However, several questions remain about the most relevant PA types and levels and the tools that should be used to better evaluate PA in elderly people. Specifically, previous cohort studies were criticized because they did not study the different PA types and did not investigate the contribution of household activities [5, 10]. The shape of the exposure-response relationship is also unclear. Xu et al. [6] found a linear association between leisure-time PA and the risk of all-cause dementia or Alzheimer’s disease (AD). However, they also reported that the AD risk curve tended to flatten for high activity levels. Therefore, it is necessary to study the form of the risk function and to determine the activity level thresholds corresponding to risk changes.

The objective of this study was to analyze the association between PA and dementia incidence in a large population-based cohort using a validated questionnaire developed specifically to explore household, transportation, leisure and sport activities in elderly people. Our secondary objectives were to determine the PA types significantly associated with dementia, to study the shape of the risk function for each activity-specific sub-score, and to identify the appropriate activity threshold values.

## Methods

### Study population

The Three-city study is a multi-site community-living cohort of 9294 participants aged 65 years and over. They were recruited between 1999 and 2001 from the electoral rolls of three French cities (Bordeaux, Dijon, and Montpellier) with the aim of studying the impact of cardiovascular factors on the risk of dementia. A standardized evaluation with a face-to-face interview and a clinical examination was carried out at inclusion and after 2 (wave W1), 4 (wave W2), 7 (wave W3), 10 (wave W4), 12 (wave W5), and 15 (wave W6) years.

The PA questionnaire was introduced at W3 (Montpellier center) and W4 (Bordeaux center) that were considered as baseline data for the current analysis. The sample included only participants who filled in the PA questionnaire during the standardized evaluation at these waves. We excluded participants who had dementia at baseline, were confined at home and did not have follow-up visits. The Ethics Committees of the University Hospital of Kremlin-Bicêtre and Sud-Méditerranée III approved the study protocol. Each participant signed an informed consent before inclusion.

### Dementia diagnosis

At baseline and at each follow-up visit, participants recruited in Bordeaux were examined by a neurologist only in the case of dementia suspicion (on the basis of an extensive cognitive and functional examination performed). In Montpellier, all participants were examined by a neurologist at each visit. Then, a panel of independent neurologists with expertise in dementia reviewed all the existing data concerning the participants with suspected dementia, and a consensus on the diagnosis of dementia was obtained according to the Diagnostic and Statistical Manual of Mental Disorders, 4th edition (DSM-IV), revised criteria and etiology. AD was classified according to the National Institute of Neurological and Communicative Disorders and Stroke and the Alzheimer’s Disease and Related Disorders Association (NINCDS/ADRDA) criteria. Prevalent dementia cases at baseline (i.e., W3 or W4) were excluded from the analyses. Participants with a diagnosis of dementia during the follow-up were considered as incident cases. The date of dementia diagnosis was defined as the midpoint between the last follow-up visit without dementia and the first follow-up visit with dementia.

### Assessment of physical activity

PA was assessed using the questionnaire developed by Voorrips and colleagues who introduced some changes in the Baecke questionnaire to make it suitable for elderly people (Voorrips questionnaire throughout the text) [11]. This questionnaire is divided in three parts: household/transportation activities, leisure time activities, and sport activities. The household/transportation activity part includes 10 questions (four to five possible scores for each item) about housework, preparing meals, shopping and transportation used (car, public transportation, bicycle, walking). The total sum (divided by 10) constitutes the first sub-score used in the present analysis. The leisure time and sport activity parts include questions on the type of activity, number of hours per week, and number of months per year. The activity types are associated with intensities that are determined according to the activity energetic costs. Sitting unloaded activities, which are mainly cognitive, were excluded from the scoring in the present study. Therefore, only standing and sport activities were considered. All leisure time and sport activities were pooled in the leisure and sport activity sub-score (intensity* number of hours per week* number of months per year). The two sub-scores progressively increase with the physical activity intensity (see Supplementary Table S1 for examples).

### Baseline covariates

Socio-demographic variables included sex, age, study center, education level (less or more than 5 years), and marital status (living alone or not).

Health status-related covariates included diabetes (antidiabetic treatment, glycemia > 7.0 mmol/L, or history of diabetes), self-reported history of cardiovascular diseases (CVD) (stroke, angina pectoris, myocardial infarction, and cardiac and vascular surgery), and presence of depressive symptoms [Center for Epidemiologic Studies Depression Scale (CES-D) scores 16–22 and ≥ 23, or antidepressant treatment (ATC code: N06A)]. Participants were also asked about the occurrence of fractures with and without hospitalization, during the 2 or 3 preceding years (all body sites). A hierarchical indicator of disability [12] was calculated by combining the Rosow and Breslau mobility scale, Lawton-Brody Instrumental Activity of Daily Living (IADL) scale, and Katz Activity of Daily Living (ADL) scale. This indicator defines four levels of disability: full independence, mild disability (only mobility restrictions), moderate disability (mobility and IADL limitation), and severe disability (mobility, IADL and ADL limitations). Participants with at least one ε4 allele were defined as APOE e4 carriers.

### Statistical analyses

Baseline characteristics were compared between included and excluded participants using the chi-square and Wilcoxon rank-sum tests.

The dementia risk was analyzed with the Cox proportional hazard regression model with delayed entry and age as time scale [13]. In addition to age, associations between baseline characteristics including physical activity and dementia incidence were adjusted for center, sex, education level, and APOE genotype (model 1). Results were expressed as hazard ratios (HR) and 95% confidence intervals (95% CI). Restricted cubic splines were generated to evaluate the adjusted dose-response [14] relationship between the two sub-scores of the Voorrips questionnaire and dementia. Different knots were tested based on Harrell’s recommendations [15], and significant splines with the lowest Akaike information criterion were chosen. The selected knots corresponded to the 5th, 35th, 65th and 95th percentiles for both sub-scores. The two median knots were used to transform the scores in classes. The first class was taken as reference in the adjusted Cox models: low activity level for household/transportation activities; no activity (score = 0) for leisure and sport activities. The proportional hazards assumption over time was verified.

The final models with classes were further adjusted for marital status, diabetes, depressive symptoms, and CVD (model 2). These potential confounders were selected on the basis of literature data and according to the strength of their association with the outcome variable (*p* < 0.1 in model 1). Additional sensitivity analyses were performed to address possible reverse causality and to assess the robustness of the results in the AD subtype. Specifically, the models were restricted to dementia occurring after the first follow-up visit (model 3), to AD only (model 4), to AD occurring after the first follow-up visit (model 5), and also to participants without IADL/ADL limitations at baseline. Interactions with sex and APOE e4 genotype were tested. All the analyses were carried out using SAS, version 9.4.

## Results

At baseline, 1301 and 1214 participants were seen at Montpellier and Bordeaux, respectively. Among these 2515 participants, 244 were not considered for the analysis because of prevalent dementia, and 115 because of they were confined at home at baseline. Among the remaining 2156 participants, 127 were excluded because they did not come to the follow-up visits. Data on PA were missing for 184 individuals and on baseline covariates for another 295 individuals. Therefore, our study sample included 1550 individuals. Compared with the analyzed sample, excluded participants were older (*p* = 0.002) and less fully independent (31.8% vs 41.2%, *p*-value< 0.0001), but with lower rates of depressive symptomatology (11.1% vs 13.3%, *p*-value = 0.04).

### Sample description

At baseline, the age of the included participants ranged between 72 and 97 years, and 63.6% were women. The median age (interquartile range (IQR)) was 80 (77–83) years. Moreover, 17% of participants had at least one APOE e4 allele, 96.8% lived at home, 76.1% had 5 years of education or more, 10.2% were diabetic, 10.7% had been hospitalized or treated for fracture, 58.8% had moderate to severe disability, and 5.8% had high depressive symptomatology. Overall, the median (IQR) follow-up time was 4.6 (3.2–7.9) years. It was 4.7 (4.0–7.9) years for participants censored without dementia, and 3.2 (2.2–4.0) years for participants with incident dementia.

During the follow-up, 117 new cases of dementia were diagnosed among whom 79 had AD. The delayed entry Cox model adjusted for age, sex, center, level of education, and presence of the APOE e4 allele (Table 1) showed that the risk of dementia was higher in participants with at least one APOE e4 allele, diabetes, moderate to severe disability, CVD history, and depressive symptoms (CES-D score > 22 or treated).

**Table 1:** Baseline characteristics and dementia incidence during the 8-year follow-up (*n* = 1550)

	Dementia status at the end of the follow-up			
	No dementia (*n* = 1433)	Dementia (*n* = 117)		
	*n* (%)	*n* (%)	HR (95%CI)^a^	*P* value^a^
Sex, female	905 (63.2)	80 (68.4)	1.08 (0.73–1.60)	0.71
Education level ≤ 5 years	337 (23.5)	33 (28.2)	1.15 (0.76–1.75)	0.50
APOE4 e4 carrier	235 (16.4)	29 (24.8)	1.87 (1.23–2.85)	0.004
Living alone	591 (41.2)	48 (41.0)	0.71 (0.47–1.06)	0.10
Diabetes	141 (9.8)	17 (14.5)	1.69 (1.01–2.85)	0.05
Hierarchical disability indicator				
Fully independent	605 (42.2)	34 (29.1)	1 (Ref)	–
Mild disability	643 (44.9)	49 (41.9)	1.16 (0.73–1.83)	0.54
Moderate to severe disability	185 (12.9)	34 (29.1)	2.55 (1.47–4.40)	0.001
Depressive symptoms				
CES-D < 16 and no treatment	1173 (81.9)	80 (68.4)	1 (Ref)	–
CES-D 16–22 and no treatment	82 (5.7)	9 (7.7)	1.84 (0.92–3.68)	0.08
CES-D ≥ 23 or treatment	178 (12.4)	28 (23.9)	2.15 (1.39–3.34)	0.001
Cardiovascular history**	227 (15.8)	28 (23.9)	1.58 (1.02–2.44)	0.04
Fractures in the past 2 or 3 years	155 (10.9)	11 (9.4)	0.75 (0.40–1.40)	0.36

Notes: *HR* Hazard Ratio, *CI* Confidence Interval, *CES-D* Center for Epidemiologic Studies Depression Scale

^a^ Cox proportional hazard model adjusted for age, center, sex, APOE genotype, and educational level

### PA sub-scores and risk of dementia

**Fig. 1:**
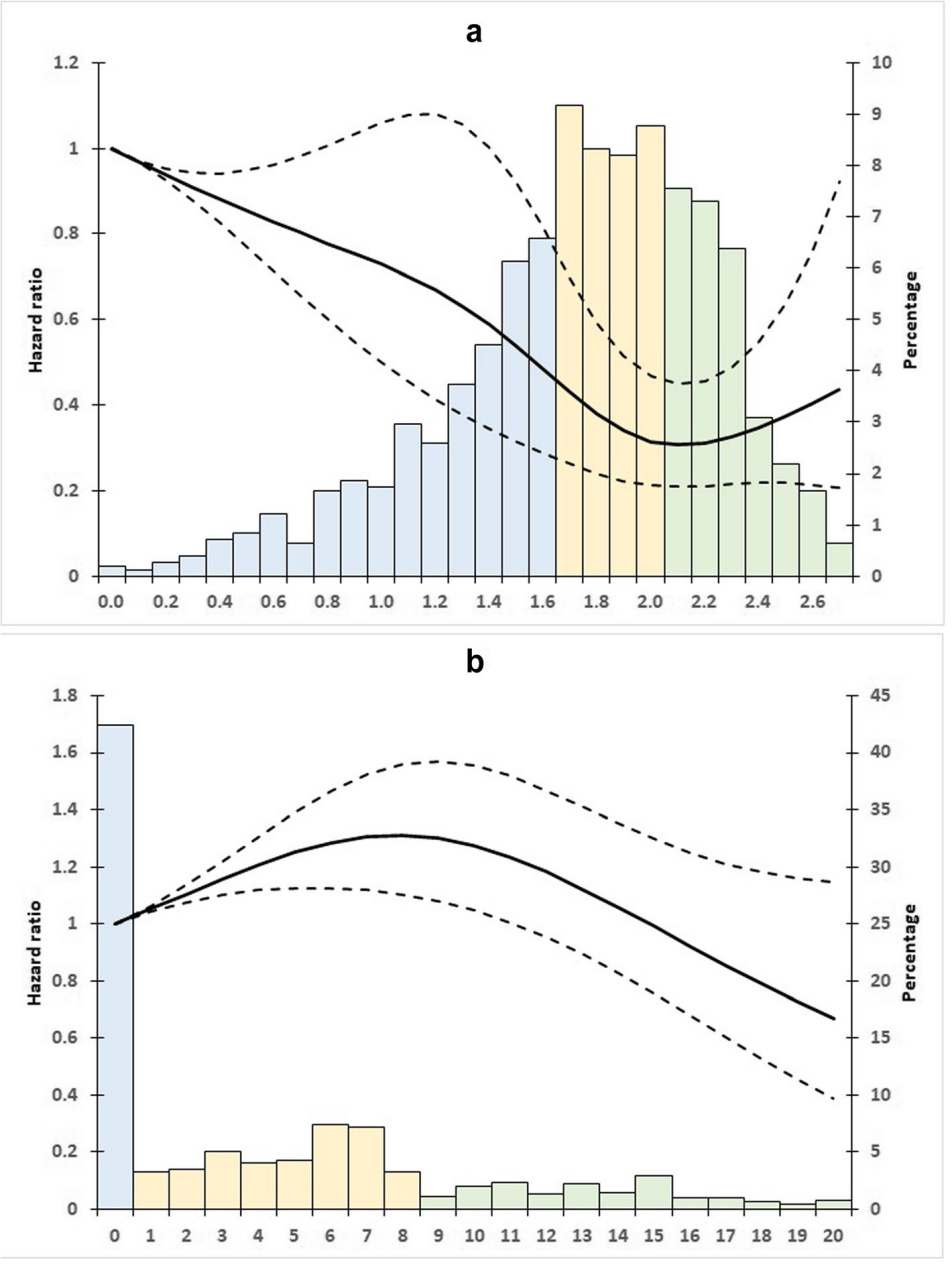
Adjusted (center, age, sex, level of education, and APOE) spline analyses of all-cause dementia risk in function of the household/transportation activity sub-scores **a** and the leisure and sport activity sub-scores **b**. Dashed lines represent the 95% confidence intervals. The score distribution is represented by the histogram in the background with the blue bars for the reference class, the yellow bars for the moderate activity class and the green bars for the high activity class

The median (IQR) sub-score was 1.8 (1.5–2.1) for household/transportation activities and 2.64 (0–6.96) for leisure and sport activities (Fig. 1). These sub-scores were equal to zero in 0.19 and 41.68% of participants, respectively. The Spearman correlation coefficient between sub-scores was 0.15, denoting a weak correlation (*p* < 0.0001). The dose-response relationships between activity type and dementia were assessed by fitting restricted cubic spline curves. The splines were significant for the household/transportation activity sub-score (*p* = 0.01), but not for the leisure and sport activity score (*p*-value = 0.33). Taking a null score as reference (no activity), the HR curve for household/transportation activities showed that the dementia risk progressively decreased with the increase of this sub-score up to 2.0 points, and then slightly increased (Fig. 1 Panel a). Conversely, the dementia risk curve for leisure and sport activities increased and then decreased (Fig. 1 Panel b).

Using the two median knots of the selected restricted cubic splines (35th and 65th percentiles) the PA sub-scores were transformed in three classes: < 1.6, [1.6;2], and > 2 for household/transportation activities, and 0,]0;8.18], and > 8.18 for leisure and sport activities. The risk of dementia according to the activity classes is summarized in Table 2. For household/transportation activities, the minimally adjusted Cox regression model (model 1) showed that the risk of dementia was lower for the second and third than the first class (HR (95%CI) = 0.53 (0.34–0.82) and HR = 0.55 (0.34–0.90), respectively). Conversely, the risk of dementia was not significantly different among the three classes for leisure and sport activities. When the model was adjusted also for marital status, diabetes, depressive symptoms, and CVD history (model 2), the dementia risk was only significantly reduced in the second class (]1.6–2.0]) of the household/transportation activity sub-score. For leisure and sport activities, results remained not significant.

**Table 2:** Association between baseline physical activity and risk of dementia

	Model 1^a^ All-cause dementia		Model 2^b^ All-cause dementia		Model 3^b^ Incident cases at the first follow-up visit excluded All-cause dementia		Model 4^b^ Alzheimer’s dementia		Model 5^b^ Incident AD cases at the first follow-up visit excluded Alzheimer’s dementia	
	n/N = 117/1550		n/N = 117/1550		n/*N* = 89/1521		n/*N* = 79/1511		n/*N* = 57/1489	
	HR (95%CI)	*p*	HR (95%CI)	*p*	HR (95%CI)	*p*	HR (95%CI)	*p*	HR (95%CI)	*p*
Household/transportation activities										
≤ 1.6	1 (Ref)	–	1 (Ref)	–	1 (Ref)	–	1 (Ref)	–	1 (Ref)	–
]1.6–2.0]	0.53 [0.34–0.82]	0.005	0.55 [0.35–0.87]	0.01	0.57 [0.34–0.97]	0.04	0.49 [0.28–0.86]	0.01	0.57 [0.29–1.10]	0.10
> 2.0	0.55 [0.34–0.90]	0.02	0.62 [0.38–1.01]	0.06	0.70 [0.40–1.24]	0.22	0.65 [0.36–1.16]	0.14	0.85 [0.43–1.68]	0.65
Leisure and sport activities										
None	1 (Ref)	–	1 (Ref)	–	1 (Ref)	–	1 (Ref)	–	1 (Ref)	–
]0–8.18]	1.18 [0.79–1.78]	0.42	1.36 [0.89–2.06]	0.15	1.12 [0.69–1.81]	0.66	1.46 [0.88–2.42]	0.15	1.08 [0.60–1.97]	0.80
> 8.18	1.03 [0.57–1.86]	0.92	1.33 [0.72–2.44]	0.36	1.17 [0.59–2.32]	0.65	1.43 [0.68–3.00]	0.35	1.15 [0.48–2.73]	0.76

n/N = number of cases/total

*HR* Hazard Ratio, *CI* Confidence Interval

^a^ Minimally adjusted Cox proportional hazard model: adjusted for age, center, sex, APOE, and education level

^b^ Multi-adjusted Cox proportional hazard model: adjusted for age, center, sex, APOE, education level, living alone, diabetes, depressive symptomatology, cardiovascular disease

The interactions between classes of both PA sub-scores and sex or APOE e4 genotype were not significant (*p*-value > 0.21 in model 2 and p-value> 0.14 in model 3).

### Sensitivity analyses

Results were similar when participants with dementia at the first follow-up visit were excluded (Table 2, model 3), and when the outcome was limited to AD (models 4 and 5). In participants without IADL/ADL limitations at baseline (*n* = 1331 and 83 incident cases of dementia), the multivariate model again indicated a significant association between dementia risk and the second class of the household/transportation activity (HR (95%CI) = 0.55 (0.32–0.95), *p* = 0.03), but not the third class (HR = 0.66 (0.37–1.19), *p* = 0.16). As PA type and intensity change with age, the associations between PA sub-score classes and dementia (or AD) risk were tested after stratification according to age (< 80 years and ≥ 80 years). All the HR were under 1, but the results (for all-cause dementia and also AD) for the second class of the household/transportation activity sub-score remained significant in the ≥80-year-old group (Table 3).

**Table 3:** Association between baseline household and transportation activities and risk of dementia stratified according to age

	Multi-adjusted model^a^ All-cause dementia		Multi-adjusted model^a^ Alzheimer’s dementia	
	HR (95%CI)	*p*-value	HR (95%CI)	*p*-value
**< 80 years**	n/*N* = 38/776		n/*N* = 23/761	
≤ 1.6	1 (Ref)	–	1 (Ref)	–
]1.6–2.0]	0.60 [0.26–1.37]	0.23	0.81 [0.27–2.46]	0.71
> 2.0	0.52 [0.22–1.23]	0.14	0.68 [0.21–2.17]	0.52
**≥80 years**	n/N = 79/773		n/*N* = 56/750	
≤ 1.6	1 (Ref)	–	1 (Ref)	–
]1.6–2.0]	0.52 [0.30–0.91]	0.02	0.38 [0.19–0.76]	0.006
> 2.0	0.69 [0.38–1.26]	0.23	0.71 [0.36–1.40]	0.32

Notes: n/N = number of cases/total

*HR* Hazard Ratio, *CI* Confidence Interval

^a^ Cox proportional hazard model adjusted for age, center, sex, APOE, education level, living alone, diabetes, depressive symptomatology, cardiovascular disease

## Discussion

This study using data from a cohort of community-dwelling participants older than 72 years of age found an inverse J-shaped association between household/transportation sub-score and risk of dementia. The greatest risk reduction (about 70%) was observed in participants with household/transportation sub-scores of about 2. In the Voorrips questionnaire, this activity level can be attained by doing some light and heavy household work with sometimes assistance, preparing warm meals 3–5 times per week, walking up 1–5 flights of stairs per day, and walking for shopping 2–4 times per week (see Supplementary Table S1). When the household/transportation sub-score was subdivided in three classes in multi-adjusted models, the risk was decreased by 45% for the moderate level (1.6–2.0 points) and by 38% for the highest level (> 2.0 points). Similar results were obtained when the analysis was restricted to AD. A reduction by 48% of the risk of dementia was also observed in the sub-sample of ≥80-year-old participants for the moderate household/transportation activity class. On the other hand, despite the decreased risk indicated by the cubic spline curve, the leisure/sport activity sub-score was not significantly associated with the disease risk. These findings suggest that in older people, maintaining a moderately high PA in everyday life is related to a lower 5-year risk of dementia.

Our study has some limitations. First, in our aged population (median = 80 years) the level of leisure and sport activities was relatively low, and 42% of them did not perform any of these activities. This may preclude detecting significant PA effects on the risk of dementia. However, this finding is representative of people belonging to this age class who are seldom involved in intense PA. When the analyses were stratified according to age, the relatively small number of dementia events (38 incident cases) also may have limited the statistical power in the 72–80 years group. Second, the low PA level could be a consequence of comorbidities that are more common at this age, and this might confound the association with dementia. However, the effect of moderate household/transportation activities persisted in the different models adjusted for a large range of covariates, including diabetes and CVD. A residual confounding may also remain because we did not have information on participants who were not confined but who could have gait and balance problems or need assistance to walk. Third, the follow-up duration was relatively short (5 years), and low PA could be an early manifestation of dementia rather than a pre-morbid risk factor. To limit this reverse causality issue, we excluded dementia at the first follow-up visit (i.e., 2 or 3 years after the baseline) or participants with IADL/ADL limitations at baseline, with unchanged results. However, our results might still partially reflect very early dementia-related behavioral changes. Finally, PA was assessed with the Voorrips questionnaire. Although self-reported PA is susceptible to information bias, it is considered a reliable method to collect data on PA for epidemiologic studies [16]. Objective methods, such as accelerometers, have known a rapid development. However, data collection and processing criteria are not always well validated, particularly in elderly people. Moreover, these methods do not give information about the activity types [17].

The strengths of this study include its multi-center longitudinal design, the sample size (*n* = 1550 elderly participants from the general population), the low attrition rate of the cohort, and the high quality of dementia screening/diagnosis. Few studies have examined the link between PA and dementia risk in very old people as we did here. PA was assessed using the Voorrips questionnaire that was validated in elderly populations and explores different types of activity. To be more clinically relevant, two sub-scores specific for two PA domains were generated, and sitting unloaded activities were excluded because they do not involve physical capacities and are mainly cognitive. To reduce the information loss these scores were analyzed as continuous measures; the exposure-response curve shape and the optimal location of the PA level thresholds were determined by a data driven method.

The literature on the effect of PA on dementia risk is abundant, but not always conclusive. Several meta-analyses have shown a protective effect of PA on dementia onset. The risk reduction for high level of PA ranges from 21 to 28% for all-cause dementia and from 26 to 45% for AD [5, 18–20]. However, results are heterogeneous, notably due to the PA type. For instance, in a systematic review that included 24 studies (among them 5 in middle-aged populations), Stephen et al. [5] reported a beneficial effect of PA in 18 of them. While the association with AD risk reduction was clear for leisure time activities, it was less obvious for occupational and commuting PA. In a meta-analysis on 44 studies (6 in middle-aged populations), Lee [10] found that high and moderate amounts of PA were related to reduced risks of all-cause, AD and vascular dementia. The author identified various activities, such as leisure time PA, regular exercise and gardening, that had a protective effect on the dementia risk. Conversely, walking was not significantly associated with the dementia risk (four studies on people aged 60 or 65 years and over). Among the 27 studies in older people, four did not find any positive effect of different PA types (swimming, walking, dancing, …) [21–23], and another one [24] detected a positive effect only for productive activities (gardening, household, …). It should be noted that sometimes, PA included also reading, watching TV, and listening to music. None of the studies clearly excluded sitting recreational activities, as we did. More studies are needed with more details on the PA type, intensity, and duration [25].

Another possible source of heterogeneity among studies is the differential subgroup effect. A more robust effect of PA on the dementia risk was found in men than in women [19]. For APOE e4 the results are conflicting with a more pronounced effect either in e4 carriers [26] or in non-carriers [27]. In our study, we did not find any significant interaction with APOE e4 and sex. The activity level was higher in women for the household activities and preparing meals but not for shopping. However, overall the benefic effect is the same for both men and women.

Little is known about the dose-response relationship between PA and dementia. In a meta-analysis that included 15 prospective studies (three in middle-aged populations), Xu et al. [6] analyzed the categorical and continuous effects of PA. First, they found that the risk of all-cause and AD dementia was reduced by 27 and 26%, respectively, in the group with the highest PA activity level compared with the group with the lowest level. Then, they observed in four studies an inverse linear dose-response relationship. Specifically, an increase in leisure time PA by 500 kcal/week was associated with a decrease of about 10% in dementia risk. Other studies examined the risk of dementia across PA quintiles and showed either a linear reduction of the risk [28], or poor evidence for a dose-response relationship [19, 27, 29]. In our study, the exposure-response curve was non-linear, and a slight risk rebound was observed for the highest household/transportation activity sub-scores. Likewise, the risk reduction was significant for the second class but the *p*-value crossed the 0.05 threshold for the third class (Table 2, model 2b). This inverse J-shaped association was already found for outcomes such as mortality [30] and stroke [14]. One explanation is that in some individuals, a high-intensity physical activity could cause sudden increase in blood pressure and hemorrhagic strokes [14], which are known to be associated with an increased dementia risk [31], but this remains to be explored. More accurate statistical methods to evaluate the continuous effect may help to determine the optimal PA level threshold. Therefore, it is difficult to formulate recommendations on PA frequency, duration and intensity because of the huge variations in PA definitions and assessment periods and methods.

Several mechanisms have been proposed to explain the role of PA in dementia. Oxidative stress has been recognized as a contributing factor to aging and the progression of multiple neurodegenerative diseases, including AD. Exercise reduces oxidative stress in the brain by inducing antioxidant enzymes. Moreover, regular PA increases the cerebral blood flow and cerebral metabolism [32]. Neurotrophic activity plays a role in modulating the brain synaptic plasticity, angiogenesis, and adult hippocampal neurogenesis through the release of neurotrophic factors (brain-derived neurotrophic factor, insulin-like growth factor, and vascular endothelial-derived growth factor) that is enhanced by exercise [33]. Animal studies have shown that exercise contributes to lowering the accumulation of beta amyloid and tau protein (2 AD hallmarks) in brain and cerebrospinal fluid [34]. All these pathways support the biological plausibility of the association between PA and reduced dementia risk.

## Conclusions

We observed a reduction of the 5-year risk of dementia in participants who performed moderately high household/transportation activities, but no significant link with leisure and sport activities, after multiple adjustments for potential confounders. This result highlights the importance of considering all physical activity types in 72 years or older people. It also illustrates the need to use specific tools for PA quantification in older individuals, because chronic diseases and frailty influence the physical functions. Our results also suggest that the household/transportation activity sub-score based on the first part of the Voorrips questionnaire, if validated in other samples, could help identifying older people at high risk of dementia. To have a broad picture, future research needs to examine all PA types, including everyday tasks, walking and the use of transportation means, and concomitantly measure PA energy and expenditure with accelerometers. Nevertheless, maintaining an active, non-institutionalized lifestyle should be promoted to preserve cognitive functions in older people.

## Supplementary information

**Additional file 1 Supplementary Table 1.** Examples of activity combinations for the household/transportation activity sub-score and the leisure and sport activity sub-score.

## Data Availability

Anonymized data will be shared by reasonable request to the 3C scientific committee.

## Abbreviations

AD: Alzheimer’s disease
ADL: Activities of Daily Living
APOE: Apolipoprotein E
ATC classification: Anatomical Therapeutic Chemical classification
CES-D scale: Center for Epidemiologic Studies Depression Scale
CI: Confidence Intervals
CVD: Cardiovascular Diseases
HR: Hazard Ratios
IADL scale: Instrumental Activities of Daily Living scale
IQR: Inter Quartile Range
PA: Physical activity
