# Supplementary material for: Physical activity types and risk of dementia in community-dwelling older people: the Three-City cohort
Source: BMC Geriatr. 2020 Apr 10;20:132. doi: 10.1186/s12877-020-01538-3 (PMC7146952; doi:10.1186/s12877-020-01538-3)
Supplement: Supplementary file 1 — Additional file 1 Supplementary Table 1. Examples of activity combinations for the household/transportation activity sub-score and the leisure and sport activity sub-score. [file 12877_2020_1538_MOESM1_ESM.docx]

**Supplementary Table 1**

**Examples of activity combinations for the household/transportation activity sub-score and the leisure and sport activity sub-score.**

| Score | **Household/transportation activity sub-score** |
| --- | --- |
| 1 | Sometimes light household work AND never heavy household work AND living alone AND taking care of 1 to 6 rooms on 1 floor AND walking up 1-5 flights of stairs per day AND never preparing meals AND using public transportation for going to the town center or for shopping AND shopping once per week |
| 1.6 (knot) | Sometimes light household work AND never heavy household work AND living with one person AND taking care of 1 to 6 rooms on 1 floor AND walking up 1-5 flights of stairs per day AND preparing meals 1-2 time per week AND walking for going to the town center or for shopping AND shopping once per week |
| 2 (knot) | Often light and heavy household work AND living alone AND taking care of 1 to 6 rooms on 1 floor AND walking up 1-5 flights of stairs per day AND preparing meals 3-5 times per week AND walking for going to the town center or for shopping AND shopping 2-4 times per week |
| 2.5 | Always light and heavy household work AND living with one person AND taking care of 1 to 6 rooms on 1 floor AND walking up 1-5 flights of stairs per day AND always preparing meals AND walking for going to the town center or for shopping AND shopping every day |
| Score | **Leisure and sport activity sub-score** |
| 5 | Gardening 8.5h/week for 8 months/year |
| 8 (knot) | Aqua-gym 1.5h/ week for 8 months/year AND walking 7.5h/week almost all year |
| 10 | Hiking 4.5h/ week for 8 months/year AND swimming 5.5h/week for 5 months/year |
